# Examining the Potential of Forest Residue-Based Amendments for Post-Wildfire Rehabilitation in Colorado, USA

**DOI:** 10.1155/2017/4758316

**Published:** 2017-02-22

**Authors:** Charles C. Rhoades, Kerri L. Minatre, Derek N. Pierson, Timothy S. Fegel, M. Francesca Cotrufo, Eugene F. Kelly

**Affiliations:** ^1^US Department of Agriculture, Forest Service, Rocky Mountain Research Station, Fort Collins, CO 80526, USA; ^2^Department of Soil and Crop Sciences, Colorado State University, Fort Collins, CO 80523, USA; ^3^Department of Crop and Soil Sciences, Oregon State University, Corvallis, OR 97331, USA; ^4^Department of Ecosystem Sciences and Sustainability, Colorado State University, Fort Collins, CO 80523, USA

## Abstract

Wildfire is a natural disturbance, though elemental losses and changes that occur during combustion and post-fire erosion can have long-term impacts on soil properties, ecosystem productivity, and watershed condition. Here we evaluate the potential of forest residue-based materials to rehabilitate burned soils. We compare soil nutrient and water availability, and plant recovery after application of 37 t ha^−1^ of wood mulch, 20 t ha^−1^ of biochar, and the combination of the two amendments with untreated, burned soils. We also conducted a greenhouse trial to examine how biochar influenced soil nutrient and water content under two wetting regimes. The effects of wood mulch on plant-available soil N and water content were significant and seasonally consistent during the three-year field study. Biochar applied alone had few effects under field conditions, but significantly increased soil pH, Ca, P, and water in the greenhouse. The mulched biochar treatment had the greatest effects on soil N and water availability and increased cover of the most abundant native plant. We found that rehabilitation treatments consisting of forest residue-based products have potential to enhance soil N and water dynamics and plant recovery following severe wildfire and may be justified where erosion risk or water supply protection are crucial.

## 1. Introduction

High-severity wildfires can cause significant, lasting impacts on forest soils and watersheds [1–4]. Near-complete combustion of vegetation and surface organic soil layers during such fires [4] exposes burned landscapes to increased soil erosion risk [5, 6]. The immediate losses of organic matter and nutrients during wildfires reduce ecosystem nutrient and carbon stocks [5, 7, 8] which may require years to decades to replenish. Decreased post-fire plant cover and nutrient demand leads to increased leaching of soluble nutrients, notably nitrogen (N), from watersheds with extensive, high-severity wildfire [3, 9]. Soil nutrient losses and physical changes that influence plant-water availability can impede post-fire revegetation [10, 11]. The frequency and extent of high-severity wildfires are projected to increase with a warming and drying climate in North American forests [12, 13] and land managers charged with maintaining forest productivity and desired watershed conditions will require effective tools for rehabilitating soils altered by severe wildfire.

Managers are working on US Forest Service land use Burned Area Emergency Response (BAER) treatments to counter immediate post-fire soil erosion losses [14]. Common BAER techniques, such as mulching [15, 16], may also assist recovery of soil productivity and speed revegetation [17] and thus contribute to post-fire restoration of ecosystem processes (i.e., soil water content and nutrient availability and retention) and native plant community composition. Mulching with woody residues is known to influence soil water, nutrient dynamics, and plant colonization of burned soils [18]. If effective, post-fire mulching would increase the utility of woody residue typically disposed of via pile burning. Forest residues are an abundant source of material; for example, in northern Colorado, there are currently more than 140,000 piles of woody residue awaiting pile burning (US Forest Service, 2012, R2, unpublished records). Developing effective uses of woody residues has the added benefit of avoiding the long-term consequences of pile burning on soils and plant composition [19].

Ecosystem restoration is enhanced by greater understanding of how disturbance and subsequent rehabilitation treatments alter soil processes and properties [20]. Biochar, a soil amendment that originates from the pyrolysis of woody forest residues and other organic feedstocks [21], is promoted for use in degraded soils due to its potential to enhance soil chemical, physical, and biological properties [22–25]. The high surface charge and microporous nature of biochar increases the pH and water-holding capacity of soils and decreases nitrate leaching [26–29]. Coupled with application of wood mulch, biochar may have potential to create favorable soil water and nutrient conditions with utility for forest road decommissioning, abandoned mine reclamation, and areas exposed by severe wildfire [30].

Experimental trials that evaluate how both established and novel rehabilitation treatments influence post-fire soil nutrient and water availability and plant establishment are required in order to develop guidelines for rehabilitating severely burned landscapes. Here we investigate the potential of wood mulch and biochar created from lodgepole pine* (Pinus contorta)* killed by mountain pine bark beetle* (Dendroctonus ponderosae)* to rehabilitate sites affected by high-severity wildfire and subsequent erosion. We evaluate individual and combined effects of mulch and biochar on post-wildfire soil and plant recovery in a replicated field study and a complementary greenhouse trial. We hypothesize that a rehabilitation treatment that combines biochar soil amendment and wood mulch surface cover will benefit soil water and nutrient retention better than either treatment applied alone. We also expect biochar to alter soil water and nutrients more under dry compared to wet conditions in a controlled greenhouse trial. These findings have implications for the current use of wood mulch for short-term, post-fire erosion control, as well as the potential use of forest residue-generated biochar and wood mulch to rehabilitate severely burned and eroded soils.

## 2. Methods

### 2.1. Study Site and Experimental Design

Research was conducted on the Arapaho-Roosevelt National Forest near Fraser, Colorado, in forests burned by the October 2010 Church's Park fire (39°56′25′′N; 105°57′00′′W). The fire burned a 200-hectare area with 17%, 30%, and 53% classified as high, moderate, and low fire severity, respectively (E. Schroder, USDA USFS BAER report). The prefire forest was dominated by mountain pine bark beetle-killed lodgepole pine with patches of quaking aspen* (Populus tremuloides)*. Bark beetles reached epidemic levels in this part of Colorado around 2000 [31]; beetle attacks increased and peaked around 2008 [32]. The burn is located between 2438 and 3200 m elevation in an area that receives ~700 mm of precipitation annually, 75% as snow. Soils are gravelly, sandy-loam Alfisols derived from colluvium and alluvium of granitic gneiss and schist parent material [33].

Post-fire rehabilitation treatments were compared in areas that burned at high severity and had visual evidence of post-fire erosion and sparse plant recovery. In June 2014, six replicate blocks of 5 × 5 m treatment plots were established on randomly selected sites that had relatively similar prefire tree species composition (>75% lodgepole pine), slope (5–15%), and aspect (south-facing). Rehabilitation treatment comparisons included (1) wood mulch, (2) biochar, (3) biochar with wood mulch, and (4) untreated burned conditions. Treatment plots were randomly assigned within each block and arranged parallel to the slope contour.

Surface amendments were designed to evaluate the potential of forest residue amendments to alter post-fire soil nutrient availability and water relations. Both woodchip and biochar treatments were created from small diameter, beetle-killed lodgepole pine. Biochar was added at an application rate of 20 t ha^−1^ and hand raked into the upper 2-3 cm of mineral soil. Woodchip mulch was applied to create a 2 cm deep surface layer, equivalent to 37 t ha^−1^. Mulch was applied above the biochar in the combined treatment. A small hand-dug trench was created upslope of the plots to reduce surface runoff reaching the experimental plots.

The study biochar was created from oven dry, lodgepole pine chips (8–10% water content) using a two-step pyrolysis process that combined an O_2_-limited step (700–750°C, <1 minute) followed by an O_2_-free step (400–550°C, 10–15 minutes). Pyrolysis was conducted by Biochar Engineering Corporation (BEC), formerly of Golden CO. Biochar consisted of 87.2% carbon (C), 1.4% 0, 0.4% N, 9.4% ash, and 1.1% water. The biochar had a pH of 9.4, surface area of 176 m^2 ^g^−1^, and total pore volume of 0.11 cm^3 ^g^−1^ [34].

### 2.2. Sampling and Analysis

We compared the effects of the rehabilitation treatments on soil nutrients, water content, and plant recovery over the course of 3 years following treatment establishment. We assessed the effects of rehabilitation treatments on plant-available soil nitrogen and potential nitrate leaching using ion exchange resin (IER) bags [35]. Resin bags were inserted 5–10 cm deep in mineral soil each fall and exchanged the following spring after snowmelt. Resin bags consisted of a 1 : 1 mixture of cation (Sybron Ionic C-249, Type 1 Strong Acid, Na^+^ form, Gel Type) and anion (Sybron Ionic ASB-1P Type 1, Strong Base OH^−^ form, Gel Type) exchange resin beads. After removal from the field, resins were extracted with a 2 M KCl solution, shaken for 60 minutes, filtered, and frozen until analysis. Nitrate and ammonium concentrations were measured by spectrophotometry using a flow injection analyzer (Lachat Company, Loveland, CO).

In August 2016, two 10 cm deep soil cores were collected and composited from each treatment plot. We measured net N mineralization and nitrification at the end of the third growing season using aerobic laboratory incubations to estimate how the treatments influenced the production of inorganic soil N [36]. Samples were kept at 4°C prior to analysis, and then roots and rocks were removed by hand and soils were well mixed. A 20 g subsample of fresh soil was placed in a 120 mL loosely capped plastic cup and wetted to 60% of field capacity [37]. Samples were incubated at 20°C and rewetted periodically. After 28 days, soils were extracted and analyzed for NO_3_^−^ and NH_4_^+^ as described above. Net transformations were calculated as follows: net mineralization = (NH_4_-N + NO_3_-N)*t*_28 d_ − (NH_4_-N + NO_3_-N)*t*_0_; net nitrification = (NO_3_-N)*t*_28 d_ − (NO_3_-N)*t*_0_ [36]. A second subsample was oven-dried at 105°C for 24 h to determine gravimetric soil water content for reporting nutrient concentrations on a dry soil mass basis. A third set of subsamples was passed through a 2 mm sieve, ground to a fine power, and analyzed for total soil C and N by Dumas dry combustion (LECO CHN 2000; St. Joseph, MI). Soil pH was analyzed in a 1 : 1 soil to deionized water slurry after one hour of agitation [38].

We also characterized water-soluble nutrients and C released from soil sampled at the end of the field study in a 5 g of soil (<2 mm size) and 100 mL of deionized water mixture. Samples were agitated for one hour, settled for 24 hours, and agitated for a second one-hour period. Samples were then filtered through 0.45 *μ*m mesh membrane filters (Millipore Durapore PVDF). Nutrient concentrations were determined by ion chromatography (Waters Co., Milford, MA) and conductivity detection with a Dionex AS12A Anion-Exchange column, an AG12A guard column, and Waters IC-Pak Cation M/D column [39]. Analysis of dissolved organic carbon (DOC) and dissolved total nitrogen (DTN) was determined by high-temperature combustion catalytic oxidation using a Shimadzu TOC-V_CPN_ total organic carbon analyzer (Shimadzu Corporation Columbia, MD). Acid neutralizing capacity (ANC) was measured by Gran titration [40] and pH and electrical conductivity (EC) were analyzed with PC Titrate sensors (Man-Tech Co.).

We measured the volumetric soil water content (0–10 cm depth) twice monthly during the 2014, 2015, and 2016 growing seasons (June–August) using a hand-held, time domain reflectometry probe (CD 620, HydroSense Campbell Scientific, Logan, UT). For each sample date, five mineral soil water values were recorded per plot beneath surface mulch or organic soil layers.

We evaluated the effect of rehabilitation treatments on plant, mineral soil, litter, and rock cover in August 2016 with a gridded point-intercept method in 1 m^2^ sample quadrats. Plant and surface cover sampling was conducted and plant nativity was classified according to the USDA NRCS Plants Database [41]. We clipped herbaceous plant biomass from 1 m^2^ quadrats and dried samples at 60°C for 48 hours.

We also isolated the effects of biochar on water content, nutrients, and chemistry of soils from the Church's Park burn under controlled greenhouse conditions. We compared two biochar levels (0 and 20 t biochar ha^−1^) under two wetting regimes (average and dry) during a six-month trial. The biochar treatment was equal to the field application rate. The dry and average wetting treatments received 4 and 8 cm of water per month, respectively. The wetting amounts were based on local long-term summer precipitation records [42]. Mineral soil (0–10 cm depth) was collected and composited from severely burned portions of the Church's Park burn. Biochar and coarsely sieved mineral soil (4 mm mesh) were thoroughly mixed and 200 g subsamples of the 0 and 20 t ha^−1^ biochar treatments were packed to a bulk density of ~1 g cm^−3^ into 500 mL planting tubes. We determined KCl-extractable-N, gravimetric water content, and pH (in 1 : 1 DI H_2_O to soil suspension and 1 : 1 0.01 M CaCl_2_ to soil suspension) at the end of the greenhouse trial. Exchangeable phosphorus and cations were extracted with Mehlich-III reagents (0.2 N CH_3_COOH, 0.25 N NH_4_NO_3_, 0.015 N NH_4_F, 0.13 N HNO_3_, and 0.001 M EDTA) [43] and analyzed by inductively coupled plasma optical emission spectrometry (Perkin Elmer Optima 7300 DV Optical Emission Spectrometer).

We compared the cumulative effects of the four field-scale rehabilitation treatments in August 2016 using one-way analysis of variance (SPSS V. 22, IBM CO, Chicago, IL). To compare treatment effects on multiple measurements of IER-N and volumetric soil water content we used a mixed model, repeated measures analysis of variance. In the greenhouse trial, biochar and wetting treatments were fixed effects in a two-way analysis of variance. Levene's statistic was used to test homogeneity of variance and data were log-transformed prior to conducting analysis of variance with that corrected normality or unequal variance. Statistical significance was assigned for *F*-test *p* values less than *α* = 0.05, and post hoc means comparisons were made on Bonferroni-adjusted *p* values.

## 3. Results

### 3.1. Field Study

#### 3.1.1. Soil Nutrients

**Figure 1:**
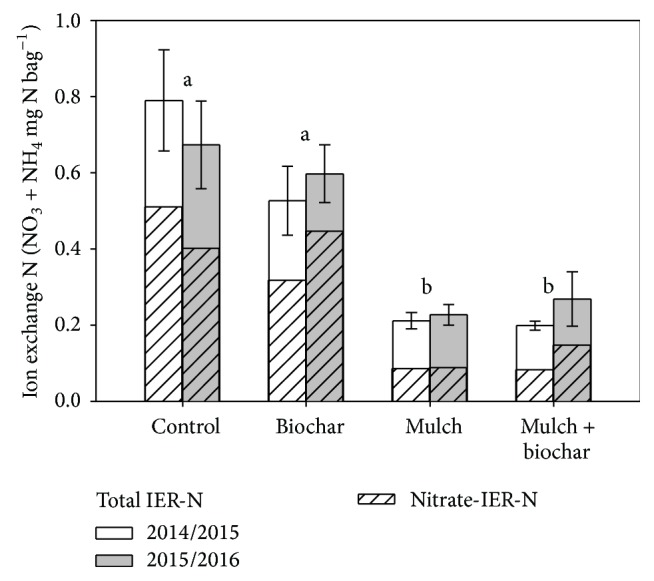
Ion exchange resin soil nitrogen comparing post-wildfire rehabilitation treatments at the Church's Park burn, Colorado. Ion exchange resin bags were installed in mineral soil (5–10 cm depth) in September and removed in early June to sample nutrients percolating in spring snowmelt. Bars show means and standard errors of six replicate treatment blocks. Letters denote significant differences among means at *α* = 0.05 level.

Mulch reduced the pool of total plant-available N measured with ion exchange resins and the proportion comprised of nitrate during the first and second snowmelt periods after treatment establishment (Figure 1). Averaged across the years, mulched plots had 70% and 80% less total and nitrate-IER-N compared to untreated, burned areas. Nitrate comprised only 41% of total IER-N in mulched plots compared to 73% of total IER-N in control plots. Biochar applied alone did not alter IER-N compared to the untreated controls. The combined treatment had no additive effect compared to mulch applied alone.

The rehabilitation treatment effects on KCl-extractable soil N measured once after three growing seasons (Table 1) agreed with the patterns we found throughout the study using IER-based measurements (Figure 1). Mulch applied either alone or in combination with biochar reduced extractable NH_4_-N and NO_3_-N; the sum of these two N forms was 75% lower in the two mulch treatments on average, compared to the untreated controls. The IER-N assays conducted during snowmelt were dominated by nitrate, and ammonium comprised most of the extractable soil N during our August 2016 samples. Net mineralization and nitrification rates measured during the lab incubation were lowest in the control plots and highest in the two mulch treatments. Total soil C, N, and pH (in water) were also the highest in the biochar plus mulch treatment and significantly different from untreated soils (Table 1). Biochar applied alone did not differ significantly from the controls but was typically intermediate between untreated and mulched plots. Similarly, the biochar plus mulch treatment did not differ significantly from the mulched plots. However, the combined treatment had the lowest measured total inorganic N and the highest total soil N, pH, and net N transformation rates (Table 1).

Nitrogen analyzed in water-soluble extracts of mineral soils collected at the end of the study further confirmed the treatment effects measured by IER-N bags (Figure 1) and soil incubations (Table 1). Mulch addition decreased water-soluble nitrate from 0.11 to 0.06 mg L^−1^ and DTN from 1.10 to 0.71 mg L^−1^, 35 and 48% reductions relative to untreated controls. The biochar plus mulch treatment did not differ from mulch applied alone. Biochar did not statistically alter either nitrate or DTN, and DOC did not differ among treated and untreated soils.

#### 3.1.2. Soil Water Content

**Figure 2:**
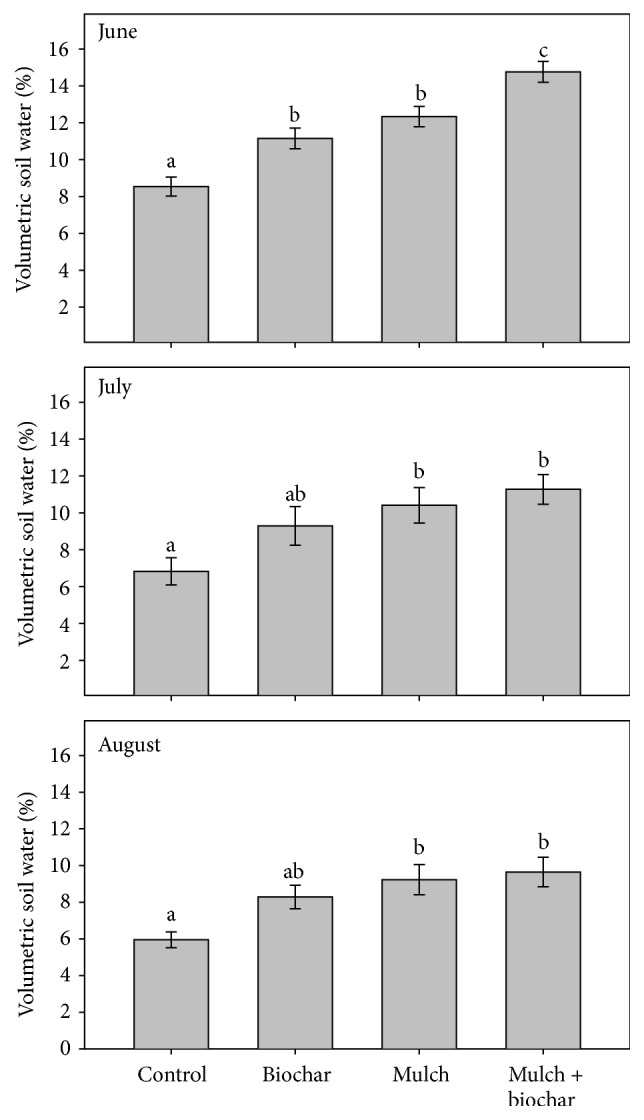
Volumetric soil water content (0–10 cm depth) under post-wildfire rehabilitation treatment at the Church's Park burn, Colorado. Soil water was measured at 6 replicate blocks of treatments, 6 times per year during 2014, 2015, and 2016. Bars show means and standard errors of six replicate treatment blocks. Letters denote significant differences among means at *α* = 0.05 level.

Individual and combined rehabilitation treatments influenced growing season volumetric water content (Figure 2). Averaged across the summer months, biochar and mulch increased soil water content 1.4 and 1.5 times above the untreated controls, respectively; the two treatments did not differ statistically. The combined treatment had the greatest effect on soil water and was 1.7 times higher than the control. Treatment differences were most pronounced in early summer when soil water content was highest; the additive effect of the biochar and mulch treatment compared to the mulch alone was greatest and statistically significant during June.

#### 3.1.3. Plant Cover

**Figure 3:**
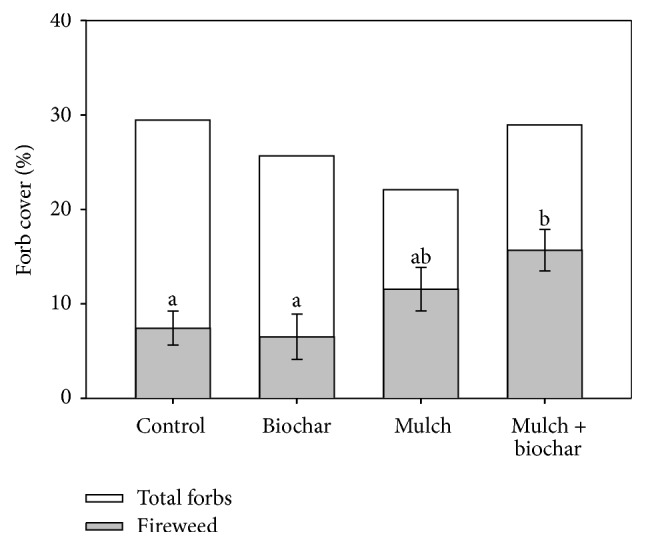
Total forb and fireweed* (Chamerion angustifolia)* cover in August 2016, the third season after post-fire rehabilitation treatment establishment at the Church's Park burn, Colorado. Bars show means and standard errors for fireweed sampled in six replicate treatment blocks. Letters denote significant differences among fireweed cover means at *α* = 0.05 level.

In summer of 2016, five growing seasons after the Church Park fire, total plant cover averaged 38% on untreated plots and consisted primarily of forbs (29%) with lesser amounts of graminoid (5%) and shrub (4%) species. The rehabilitation treatments had no general effects on the cover of plant functional groups (i.e., forbs, graminoids, and shrubs), but had specific effects on the most common species. Fireweed* (Chamerion angustifolia)*, the species with the highest cover in the study area (~25% of total plant cover), had significantly higher cover in both mulch treatments (Figure 3) relative to untreated plots. Fireweed covered 16% of mulched biochar plots, more than half the total forb cover in that treatment. Fireweed biomass was also highest in mulch-treated plots. It represented 43% of total annual biomass production in the mulch plus biochar treatment, nearly twice the proportion found in control plots (22%). In contrast to fireweed, the cover of the second-most common forb,* Gayophytum diffusum *spp.* parviflorum* (spreading groundsmoke), was inhibited by the mulch treatments. Mulching caused similar, but not statistically significant, declines in cover of other common forb groups (Asteraceae) and species* (Lupinus argenteus)*. No species responded to the biochar treatment.

### 3.2. Greenhouse Study

At the end of our six-month greenhouse trial, Church's Park soil mixed with 20 t ha^−1^ of biochar had higher concentrations of most exchangeable nutrients and higher soil pH and gravimetric water (Table 2). Soil pH was 0.4 units higher in the treated soils, nitrate was 6 times higher, and exchangeable P and K were both more than 1.2 times higher. Soil pH measured in a weak salt solution was 1 pH unit lower than that measured in DI water, but it showed the same magnitude biochar treatment effect (0.4 units) and no effect of watering. Similar to what we found in the field plots (Table 1), this indicates that biochar had no ionic strength-related influence on pH [38]. Responses to biochar addition varied significantly with watering regime only for gravimetric water content and exchangeable P; in both cases, biochar had a greater effect under the drier soil conditions (Table 2). Nitrate and K were lower in wetter treatments, independent of the biochar treatment. Nitrate was the dominant exchangeable N form at the end of the incubation, comprising over 90% in the soils containing biochar (Table 2).

## 4. Discussion

### 4.1. Post-Wildfire Rehabilitation

This project compared rehabilitation treatments designed to improve soil conditions and speed native plant recovery after high-severity wildfire. The treatments, in particular those including mulch, had significant effects on soil water and nutrient relations (Figures 1 and 2 and Tables 1 and 2) in ways expected to influence post-fire plant establishment and growth. For example, exposed soil cover remained high (60%) on untreated sites at the end of the study, compared to the extensive woody residue cover of the mulched plots (75%), so it was surprising that total plant cover did not differ. However, biochar plus mulch significantly increased cover of the most abundant forb species (*C. angustifolia*) and of various longer-lived woody species. Our findings demonstrate species-specific responses to fire and rehabilitation and underscore the need to consider how plant life history traits (e.g., annual, perennial, and sprouter) will contribute to post-disturbance plant community composition [44]. In the absence of any rehabilitation efforts, total plant cover increased from 13 to 40% over the course of the study (2013–2016). The unassisted plant recovery highlights the resilience of ecosystems to severe wildfire and the need to evaluate restoration in the context of natural ecosystem dynamics.

### 4.2. Biochar

Biochar has been widely promoted as a soil amendment to improve plant nutrient and water availability [45], yet recent research syntheses [45–49] document positive, neutral, and negative responses to biochar additions. These highly variable results caution against broad application of biochar. Biochar includes a large variety of compounds, and the need to better characterize how variation in feedstock and pyrolysis conditions influence its chemical and physical properties is well understood [30]. Pine biochar did not negatively impact soil properties or plant cover in our study and had positive effects under some conditions. The stable forms of C found in biochar will also contribute to long-term soil C sequestration [50]. However, we found little evidence that biochar when added alone represents an effective short-term rehabilitation treatment for soils altered by severe wildfire.

Our greenhouse trial demonstrated substantial effects of biochar on soil nutrient and water availability in a controlled environment. The 20 t ha^−1^ biochar treatment increased soil water, pH, nitrate, P, Ca, and K (Table 2), typically to a greater extent under the dry watering regime. When added to field plots, biochar increased volumetric soil water significantly during moist, early summer conditions and marginally during later, drier periods (Figure 2). General agreement between our field and greenhouse studies confirms the widely reported [49], though not universal, positive effect of biochar on soil water content [51]. In contrast, it was surprising that biochar had differing effects on soil nitrate in the greenhouse and field trials (Tables 1 and 2). The limited general effect of biochar in the field study agrees with feedstock comparisons reporting that pine-derived biochar has fewer consistent benefits than hardwood feedstock. Greenhouse trials help isolate ecological factors or processes but do not capture the complexity of field conditions [30]. The challenge of incorporating biochar into rocky, forest soils and possible losses from field plots may have augmented differences between these aspects of our research.

### 4.3. Wood Mulch

Like numerous other studies [10, 52–54], we found that wood mulch had substantial effects on soil and vegetation. Findings from the Church's Park burn demonstrate a potential use for this woody residue to rehabilitate soils altered by severe wildfire and to mitigate erosion [6, 55] and water quality concerns [3]. We found that wood mulch consistently increased soil water content and reduced plant-available soil N pools that are typically elevated in burned soils [7, 56]. Inorganic soil N declines as it is immobilized within wood mulch or similar high C, low N soil amendments [53, 57]. The low IER nitrate we reported in mulched plots (Figure 1) is similar to reduced post-fire nitrate losses measured under wood mulch elsewhere [58, 59]. In another study in Colorado forests, thick mulch reduced nitrate to a greater extent than thin mulch (7.5 versus 15 cm [60]). The relatively thin mulch at the Church's Park fire (2 cm) was sufficient to consistently reduce nitrate leaching potential and increase soil water. Higher total soil C and N and net N mineralization and nitrification rates measured under the mulch treatments indicates that soil productivity and N supply have improved relative to untreated Church's Park soils.

Where applied for restoration of burned soils, mulch can both stimulate and suppress native plants [17, 18, 58, 61]. At Church's Park, we found that it increased cover of the most abundant forb species, decreased cover of a few less-common species, but had no net effect on total plant cover. Other studies have shown similar, species-specific patterns. Cover of a large-seeded, aggressive grass* (Elymus trachycaulus)* that was seeded in burned soils, was stimulated by 5 cm of wood mulch [58], but various other grass species were not. A deeper mulch application (10 cm) suppressed establishment of a diverse species mix, designed to rehabilitate slash pile burn scars [18]. As part of mechanical fuel reduction operations in Colorado conifer forests, masticated wood mulch had generally positive effects on understory plant cover the first decade after treatment [62]. Mulch does not favor all plant species, but we found that it helped support an adequate plant density to reduce erosion and leaching and also permit establishment of additional herbaceous, shrub, and tree species.

### 4.4. Mulch + Biochar

For most the factors we measured, biochar combined with a wood mulch layer had the greatest effects, consistently exceeding those of biochar alone. This agrees with findings that biochar treatments are enhanced by addition of fertilizer, microbial inocula, or organic amendments [47, 63, 64]. Lower IER-N and extractable-N together with higher total N and C, soil water, and net N transformation rates suggest that N demand and N supply have become recoupled and similar to prefire conditions. The abundance of native shrubs and high forb cover in the biochar plus mulch treatment is indicative of greater nutrient demand, litter inputs, and soil stabilization. Given our initial findings, the combined treatment appears likely to favor continued establishment of plants adapted to the soil nutrient and microclimatic and plant competition levels characteristic of the uniform soil organic layers found in mature forests [65–67].

### 4.5. Management Implications

The increase in understory cover that occurred during this study indicates that unassisted post-fire recovery is well underway. Nevertheless, bare soil remains high and shrub cover remains negligible (<4%) in the control plots, and tree regeneration was absent in the study treatments and surrounding areas. The timing of the Church's Park fire, 5–8 years after bark beetles killed most of the overstory lodgepole pine, has delayed forest regeneration compared to the profuse and rapid establishment and growth of tree seedlings in nearby unburned and salvage-logged stands [68]. The post-burn soil nitrate pulse should decline with time [18, 69], but persistent shifts in plant demand and soil N cycling may prolong the effects of this fire. Elsewhere in Colorado, post-fire stream nitrate remained 100 times above background for more than five years in watersheds that have had slow forest regeneration after extensive, severe wildfire [3]. In the context of natural recovery, extreme, prolonged, or socially unacceptable wildfire effects may justify rehabilitation in fire-adapted ecosystems.

Immediate post-fire treatments aimed at avoiding soil nutrient and organic matter losses have the combined benefit of supporting ecosystem productivity while limiting nutrient export and potential water quality degradation. Our work began 2 years after the Church's Park fire; the treatment effects may have been greater had rehabilitation been conducted within months of the fire, as is typical of the BAER program [14]. Wood mulch is gaining favor for post-fire rehabilitation since it persists longer than agricultural mulch and is commonly available [10, 15, 55, 59]. Biochar is becoming more widely available for forest application [70] and forest restoration [30, 66, 67]. Application of wood and straw mulch in post-fire forest settings is commonplace, but techniques for handling and applying biochar developed for agriculture will require modification to function under the emergency post-fire response conditions or complex rocky terrain that typify forest landscapes. Rehabilitation efforts, such as our biochar plus mulch treatment, could augment the value of emergency erosion mitigation efforts by maintaining or restoring soil productivity and by contributing to long-term soil C sequestration [50]. Future research to track longer-term effects of the treatments and to evaluate ways to integrate them into emergency post-fire actions will expand on these findings and help develop effective, practical, and persistent rehabilitation treatments.

## Figures and Tables

**Table 1:** Soil properties and net N incubations 3 years after establishment of rehabilitation treatments at the Church's Park fire, Colorado. Treatments include lodgepole pine-derived biochar and wood chip mulch, their combination, and untreated, severely burned soils. Data are means and standard error in parentheses (*n* = 6 blocks of 5 × 5 m study plots).

	Control	Biochar	Mulch	Mulch + biochar	*F*	*p*
pH_water_	5.7 (0.13)^a^	5.8 (0.09)^ab^	5.7 (0.13)^a^	6.4 (0.20)^b^	4.6	0.014
pH_salt_	5.3 (0.16)	5.1 (0.10)	5.1 (0.17)	5.7 (0.20)	2.7	0.073
NH_4_-N (mg N/kg)	1.6 (0.37)^a^	1.2 (0.52)^ab^	0.6 (0.15)^bc^	0.2 (0.05)^c^	3.5	0.032
NO_3_-N (mg N/kg)	0.4 (0.11)^ab^	0.5 (0.18)^a^	0.1 (0.03)^b^	0.1 (0.05)^b^	4.3	0.015
Total N (g N/kg)	0.8 (0.04)^a^	0.7 (0.07)^ab^	0.8 (0.07)^ab^	1.0 (0.05)^b^	6.6	0.002
Total C (g N/kg)	17.4 (1.7)^a^	19.3 (1.6)^ab^	20.2 (1.6)^ab^	24.5 (1.3)^b^	3.5	0.031
C : N	21.4 (1.60)^a^	29.6 (1.84)^b^	24.3 (1.13)^ab^	23.8 (0.56)^a^	6.3	0.003
Net mineralization (mg N/kg/28 d)	−0.7 (0.48)^a^	−0.4 (0.47)^ab^	0.4 (0.07)^bc^	0.6 (0.17)^c^	3.5	0.032
Net nitrification (mg N/kg/28 d)	−0.2 (0.10)^a^	−0.3 (0.13)^a^	0.1 (0.05)^b^	0.2 (0.11)^b^	5.1	0.007

Letters denote significant differences among treatment means at *α* = 0.05 level.

**Table 2:** Soil properties after a six-month greenhouse trial with lodgepole pine-derived biochar and soil from areas affected by the Church's Park fire, Colorado. Data are means and standard error in parentheses (*n* = 6 per treatment).

		Biochar	Watering regime		*p* values		
		t/ha	Dry	Wet	Biochar	Water	Char × water
Gravimetric water content	(%)	0	12.1 (0.7)	20.5 (0.6)	<0.001	<0.001	<0.001
		20	21.7 (1.0)	23.0 (0.9)			
pH_w_	—	0	5.6 (0.05)	5.6 (0.05)	<0.001	0.570	0.731
		20	5.9 (0.06)	6.0 (0.08)			
pH_salt_	—	0	4.5 (0.07)	4.6 (0.03)	<0.001	0.128	0.401
		20	4.9 (0.04)	5.0 (0.06)			
NO_3_-N	(mg/L)	0	1.8 (0.3)	0.8 (0.2)	<0.001	0.002	0.297
		20	7.9 (0.7)	5.8 (0.5)			
NH_4_-N	(mg/L)	0	0.3 (0.1)	0.5 (0.1)	0.524	0.231	0.441
		20	0.3 (0.1)	0.3 (0.1)			
P	(mg/L)	0	36.0 (1.3)	38.2 (1.0)	<0.001	0.520	0.026
		20	49.5 (0.6)	45.7 (1.9)			
K	(mg/L)	0	23.6 (1.2)	22.5 (0.9)	<0.001	0.009	0.115
		20	30.5 (0.9)	26.2 (0.9)			
Mg	(mg/L)	0	16.6 (1.6)	16.6 (1.7)	0.253	0.883	0.888
		20	18.7 (1.6)	18.2 (1.5)			
Ca	(mg/L)	0	99.5 (4.0)	95.7 (3.7)	0.001	0.166	0.785
		20	112.2 (2.7)	106.6 (2.8)			
